# Measurement of Neutron Decay Parameters—The abBA Experiment

**DOI:** 10.6028/jres.110.058

**Published:** 2005-08-01

**Authors:** W. S. Wilburn, J. D. Bowman, G. S. Mitchell, J. M. O’Donnell, S. I. Penttila, P.-N. Seo, J. R. Calarco, F. W. Hersmann, T. E. Chupp, T. V. Cianciolo, K. P. Rykaczewski, G. R. Young, R. T. De Souza, W. M. Snow, D. Desai, G. L. Greene, R. K. Grzywacz, E. Frlez, D. Pocanic, T. R. Gentile, V. Gudkov, G. L. Jones

**Affiliations:** Los Alamos National Laboratory, Los Alamos, NM 87544; University of New Hampshire, Durham, NH 03824; University of Michigan, Ann Arbor, MI 48109; Oak Ridge National Laboratory, Oak Ridge, TN 37831; Indiana University, Bloomington, IN 47408; University of Tennessee, Knoxville, TN 37996; University of Virginia, Charlottesville, VA 22904; National Institute of Standards and Technology, Gaithersburg, MD 20899; University of South Carolina, Columbia, SC 29208; Hamilton College, Clinton, NY 13323

**Keywords:** neutron beta decay, weak interactions

## Abstract

We are developing an experiment to measure the correlations *a*, *A*, and *B*, and the Fierz interference term *b* in neutron decay, with a precision of approximately 10^−4^. The experiment uses an electromagnetic spectrometer in combination with two large-area segmented silicon detectors to detect the proton and electron from the decay in coincidence, with 4π acceptance for both particles. For the neutron-polarization-dependent observables *A* and *B*, precision neutron polarimetry is achieved through the combination of a pulsed neutron beam, under construction at the SNS, and a polarized ^3^He neutron polarizer. Measuring *a* and *A* in the same apparatus provides a redundant determination of *λ* = *g_A_/g_V_*. Uncertainty in *λ* dominates the uncertainty of CKM unitarity tests.

## 1. Introduction

Because free neutron decay is unencumbered by the many-nucleon effects present in all other nuclear decays, measurements of the parameters that describe neutron decay can be related to the fundamental weak couplings in a straightforward fashion. Within the framework of the standard model, neutron decay can be used to determine the weak vector coupling constant *g_V_* in a fashion that is relatively free of theoretical uncertainties. In combination with measurements of the weak-coupling constant in muon decay, *g_V_* provides a value for the *V_ud_* which can be compared with high energy experiments in the strange quark sector to provide important information regarding the completeness of the three-family picture of the standard model through a test of the unitarity of the CKM matrix [1]. Such a comparison provides a powerful and quite general test of the standard model. A convenient point of departure for the discussion of the physics of neutron decay is the expression for the polarized neutron decay rate as a function of electron energy *E_e_* given by [2]

$$dW\propto \frac{1}{\tau_{n}}F(E_{e})\left[1+a\frac{p_{e}\cdot p_{v}}{E_{e}E_{v}}+b\frac{m_{e}}{E_{e}}+A\frac{\sigma_{n}\cdot p_{e}}{E_{e}}+B\frac{\sigma_{n}\cdot p_{v}}{E_{v}}\right],$$ (1)

where *τ*_n_ is the neutron lifetime, ***p***_e_ and ***p***_ν_ are the outgoing electron and neutrino momenta, ***σ***_n_ is the neutron spin, and *E*_ν_ is the neutrino energy. *F*(*E*_e_) in the familiar beta energy spectrum.

We propose to perform a high-precision measurement of the correlations in neutron decay employing a new method, which provides a substantive advance over previous measurements. Unlike previous measurements [3–16] which are capable of measuring only one correlation coefficient such as *A* (the correlation between the neutron spin and the decay electron momentum), our experiment will provide a complete set of correlations including not only *A*, but also *B* (the correlation between the neutron spin and the decay neutrino), *a* (the correlation between the neutrino momentum and the decay electron momentum), and the electron energy spectral distortion term *b*. In the standard model, these four parameters are highly correlated, depending on only one free parameter *λ* = *g_A_/g_V_*. If physics beyond the standard model is included, the relationship between these coefficients is more complex and depends upon the details of the new physics. Sensitivity estimates based on the fluxes in HFIR and SNS indicate that we can expect up to an order of magnitude improvement in each of these coefficients and in the case of *b*, the first measurement ever. The spectrometer and methods we have developed provide means to control systematic errors never before available in the study of neutron beta decay.

The new experiment includes a number of novel features. Several of these address known problems in previous neutron decay correlation experiments.

- Neutrons will be polarized using a nuclear spin polarized helium-3 transmission cell. The use of pulsed neutrons with such a cell has been shown to provide an exceedingly robust determination of the neutron polarization.
- Decay electrons and protons will be detected in coincidence. Realistic tests have shown that this will significantly reduce backgrounds.
- Both electrons and protons will be detected in the same silicon detectors. The use of a strong magnetic field makes this a 4π detector for both electrons and protons.
- Proton time-of-flight will be exploited to provide information about the axial component of the proton momentum. This greatly enhances the experimental sensitivity to *a* and provides an important check on systematic effects.
- The neutron decay volume is defined by magnetic fields. The neutrons do not interact with any matter in the decay volume.
- The decay particles, *e* and *p*, do not interact with apertures, grids, or windows. They see only *E* and *B* fields and the silicon detectors.

The experiment will be performed in two phases. Phase one, starting in 2007, will use an un-polarized continuous cold neutron beam from HFIR. In this phase, the correlations *a* and *b* will be measured. Phase two will be performed at the SNS with a polarized pulsed cold neutron beam, where *B* and *A* will be determined as well as *a* and *b*. The same decay spectrometer will be used in both phases. Phase two could begin in 2008 when high-power operation begins at SNS.

## 2. Description of the Proposed Experiment

**Fig. 1:**
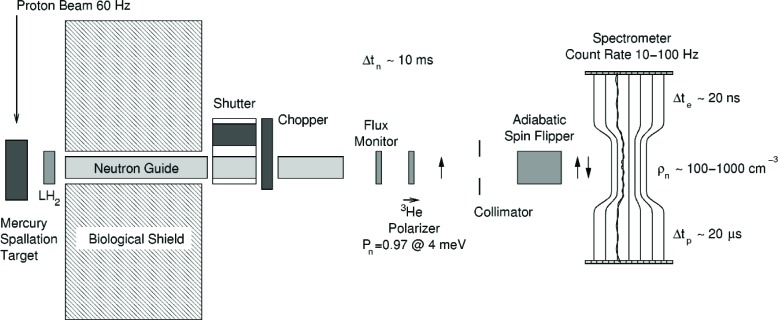
The conceptual design for the abBA experiment, shown in the SNS (polarized neutron beam) configuration.

There are three main components to our apparatus: 1) the decay spectrometer, 2) the neutron polarizer, and 3) the neutron source. These components are shown schematically in Fig. 1.

**Fig. 2:**
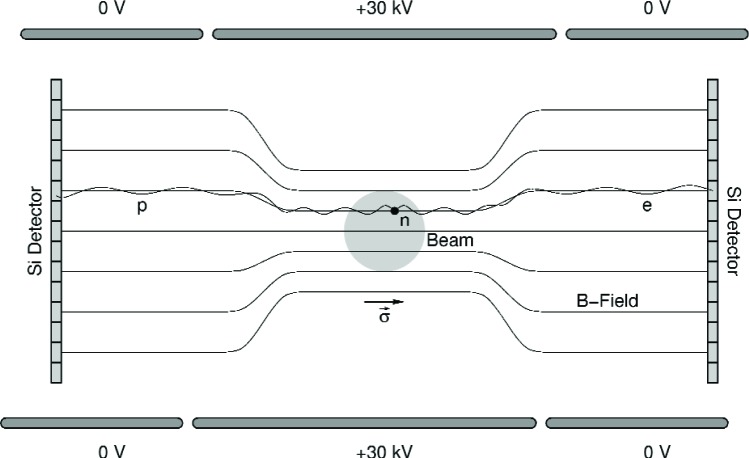
The conceptual design for the spectrometer showing the most important elements (not to scale). The beam direction is into the page.

The decay spectrometer is a highly efficient 4π neutron decay detector sensitive to electron-proton coincidences. A schematic of the detector is shown in Fig. 2. The essential components of the detector are:

### Superconducting Magnet

The decay volume (roughly 40 cm^3^) is in a very uniform magnetic field of 3.2 T. The axis of the field determines the axis of neutron polarization as well as the projection axis for the decay particle detectors. Charged decay particles follow the magnetic field lines in helical orbits. Beyond the decay region, the field decreases to 1 T. This field decrease serves to longitudinalize the charged particle trajectories and thus significantly reduce the systematic effects of electron Penning trapping. This field change also serves as a Θ-pinch magnetic mirror to mitigate the effects of electron backscattering from the detectors.

### High Voltage Electrodes

Because the maximum proton energy is only ≈750 eV, the decay volume is within a tubular electrode held at ≈30 kV. As the protons drift toward the end of the tube they are accelerated to and gain enough energy to be detected in a Silicon detector. The aspect ratio of the tube is selected to insure that the *E*-field in the decay volume is sufficiently small so that systematic effects are not important. The neutrons enter the electrode through long tubular arms to allow a windowless system.

### Silicon Detectors

Electrons *and* protons are detected in the *same segmented silicon detectors*. There are no grids and no secondary emission foils. The strong magnetic field insures that the only low energy particles that can strike the detector come from the decay volume which is at ultra-high-vacuum. Detection efficiency is essentially 4π. The detectors have an active thickness of ≈2 mm which is sufficient to insure 100 % electron energy deposit. The detectors have a thin gold dead layer (≈20 µg/cm^2^) to insure the entrance energy loss is small (≈5 keV). The detectors will be ≈10 cm diameter wafers with approximately 100 active pixels on each detector. Detectors and preamps will be cooled to LN_2_ temperature and each pixel area will be 1 cm to 2 cm. This will allow a trigger threshold of ≈5 keV [17].

### Detector Electronics and DAQ

The identification of backscattered elections requires relatively fast timing (approximately few nanoseconds). Because the energy deposit of a single particle can span more than one pixel, we require detector electronics that can provide flexible event identification. Our DAQ will be based on a 12 bit, 100 MHz Digital Signal Processor.

A decay event is identified by a delayed coincidence between a fast electron (TOF≈10 ns) and a slow proton (TOF on the order of tens of microseconds). For each decay event, the following information can be determined: total electron energy, sign of axial component of the electron momentum, sign of axial component of the proton momentum, and magnitude of the axial component of the proton momentum [18].

Our approach to neutron polarization relies on the use of nuclear spin polarized ^3^He gas cells as a neutron spin filter. ^3^He has an extremely strong spin dependent capture cross section with essentially all capture in one spin state. The neutron polarization following transmission through a polarized ^3^He cell will be given by

$$P_{n}(v)=\tanh[P_{3}Nl\sigma (v)],$$ (2)

where *P*_n_(*v*) is the neutron polarization, *P*_3_ is the ^3^He polarization, *N* is the ^3^He number density in the cell, *l* is the ^3^He cell thickness, *σ*(*v*) is the unpolarized capture cross section at neutron velocity *v*.

By exploiting the simple and well understood interaction between neutrons and ^3^He at low energy, the neutron polarization can be accurately determined [19]. At a pulsed source, time of flight (TOF) for cold neutrons makes it is possible to relate the neutron polarization to the TOF.

$$P_{n}(v)=\tanh\left(\frac{P_{3}Nl\sigma (v_{0})v_{0}}{L}t\right)=\tanh(t/\tau ),$$ (3)

where *L* is distance from the source to the experiment and *t* is the TOF. In the above, *τ* is *the* single instrumental parameter that needs to be determined to extract an accurate polarization. For example, in the neutron-spin/beta-momentum correlation experiment, the measured asymmetry *A*_exp_ = *AP*_n_. Thus the fundamental asymmetry will have the same, well understood, parametric dependence on TOF and can be extracted by a fit to the asymmetry data. This procedure provides an *in situ* determination of the polarization of the neutrons that actually undergo decay in the apparatus.

When it becomes operational in early 2005, the new cold source at the High Flux Isotope Reactor (HFIR) will be the most intense cold neutron source in the United States. The end position of HFIR Cold Guide 4 (CG4) will be available for this project. ORNL intends to construct a specialized, converging neutron guide on the end of CG4 to provide the highest possible neutron density at the decay-detector volume. Beamline simulations indicate that the neutron density at the decay volume will be ≈11 000 cm^−3^ [20]. In the projected decay volume of ≈40 cm^3^, we would expect a rate of ≈500 s^−1^.

Our method of absolute neutron polarimetry using ^3^He requires access to a pulsed neutron source. Currently Flight Path 12 (FP12) at the Manuel Lujan Center at Los Alamos is the only cold, pulsed, neutron beam in the world that is available for neutron nuclear physics. In ≈2008 a new beamline Flight Path 13 (FP13) at the SNS will become operational and be available for nuclear physics experiments. At the SNS, we expect a polarized neutron density of density of ≈4000 cm^−3^ and an event rate of ≈180 s^−1^.

## 3. Conclusion

We are developing an experiment to measure the neutron decay parameters *a*, *b*, *B*, and *A* with greatly improved precision. The experiment uses the time-of-flight characteristics of a pulsed cold neutron beam combined with the properties of a polarized ^3^He neutron polarizer to monitor the neutron polarization *in situ*. Large-area silicon detectors are used to detect both proton and electron from the decay in coincidence, greatly reducing backgrounds compared to previous singles experiments. We plan to run the experiment first at the HFIR reactor, to measure the polarization-independent parameters *a* and *b*, and then at SNS to measure all four parameters.

## References

[1] Groom DE (2000). Europhys J C.

[2] Jackson JD, Treiman SB, Wyld HW (1957). Phys Rev.

[3] Stratowa C, Dobrozemski R, Weinzierl P (1978). Phys Rev D.

[4] Erozolimsky BG, Frank AI, Mostovoy YA, Azumanov SS, Voitzik LR (1979). Yad Fiz.

[5] Bopp P (1986). Phys Rev Lett.

[6] Bopp P (1988). Nucl Instrum Methods A.

[7] Erozolimsky BG (1990). Yad Fiz.

[8] Erozolimsky BG (1991). Phys Lett B.

[9] Kuznetsov IA (1995). Phys Rev Lett.

[10] Schreckenbach K (1995). Phys Lett B.

[11] Abele H (1997). Phys Lett B.

[12] Liaud P (1997). Nucl Phys A.

[13] Yerozolimsky B, Kuznetsov I, Mostovoy Yu, Stepanenko I (1997). Phys Lett B.

[14] Serebrov AP, Kuznetsov I (1998). JETP Lett.

[15] Abele H (2002). Phys Rev Lett.

[16] Byrne J (2002). J Phys G.

[b17-j110-4wil2] 17P.-N. Seo et al., this Special Issue.

[b18-j110-4wil2] 18J. D. Bowman et al., this Special Issue.

[b19-j110-4wil2] 19S. I. Penttila et al., this Special Issue.

[b20-j110-4wil2] 20R. Mahurin et al., this Special Issue.

